# Addressing the heterogeneity in liver diseases using biological networks

**DOI:** 10.1093/bib/bbaa002

**Published:** 2020-03-23

**Authors:** Simon Lam, Stephen Doran, Hatice Hilal Yuksel, Ozlem Altay, Hasan Turkez, Jens Nielsen, Jan Boren, Mathias Uhlen, Adil Mardinoglu

**Affiliations:** Centre for Host-Microbiome Interactions, Faculty of Dentistry, Oral & Craniofacial Sciences, King’s College London, London, SE1 9RT, United Kingdom; Science for Life Laboratory, KTH - Royal Institute of Technology, Stockholm, SE-17121, Sweden

**Keywords:** Systems biology, Computational biology, Liver metabolism, Genome-scale metabolic model, Integrated network, Omics integration

## Abstract

The abnormalities in human metabolism have been implicated in the progression of several complex human diseases, including certain cancers. Hence, deciphering the underlying molecular mechanisms associated with metabolic reprogramming in a disease state can greatly assist in elucidating the disease aetiology. An invaluable tool for establishing connections between global metabolic reprogramming and disease development is the genome-scale metabolic model (GEM). Here, we review recent work on the reconstruction of cell/tissue-type and cancer-specific GEMs and their use in identifying metabolic changes occurring in response to liver disease development, stratification of the heterogeneous disease population and discovery of novel drug targets and biomarkers. We also discuss how GEMs can be integrated with other biological networks for generating more comprehensive cell/tissue models. In addition, we review the various biological network analyses that have been employed for the development of efficient treatment strategies. Finally, we present three case studies in which independent studies converged on conclusions underlying liver disease.

## Introduction

The global burden of complex diseases is rising and such diseases cause millions of deaths each year worldwide, proving to be a widespread issue for not only individuals and healthcare systems but also researchers and clinicians alike. Liver diseases, including non-alcoholic fatty liver disease (NAFLD), non-alcoholic steatohepatitis (NASH), liver cirrhosis and hepatocellular carcinoma (HCC), account for over 3 million deaths per year worldwide, with 1.3 billion adults currently overweight with a 25% lifetime risk of NAFLD [1]. To date, there is no universal therapy for NAFLD and NASH. Instead, patients reduce personal risk factors by implementing lifestyle changes, such as dieting and exercise, or therapeutic solutions, such as insulin sensitisers (e.g. metformin), antioxidants (e.g. vitamin E) and cholesterol-lowering agents (e.g. statins) [2]. Although somewhat successful, the underlying mechanisms of action of many of these therapeutics and potential ramifications of their use continue to be poorly understood, especially on the molecular and cellular levels.

HCC patients display genetic, transcriptomic, proteomic, metabolomic, fluxomic and/or metagenomic heterogeneity—that is to say, no two cases are identical. However, all cases are broadly classified as HCC. This heterogeneity in complex diseases thus implies that personalised therapies are not only desirable, but necessary to effectively treat HCC while minimising side-effects.

As reviewed elsewhere [3], systems biology approaches have been successfully employed to study interactions in complex disease on multi-omics levels. It has already proven invaluable for the discovery of biomarkers for the stratification of patients of complex diseases as well as to identify prognostic markers and potential therapeutic targets [4–6], and has thus informed personalised therapy for the patients of heterogeneous diseases. The advent of the falling cost of multi-omics profiling has meant that investigators today have a wealth of biological and clinical data available to them for the generation of both general [7] and personalised [8, 9] biological networks. This, along with growing computational power and libraries of multi-omic tools, means that investigators are now able to perform more *in silico* perturbations and simulations to obtain a global, systems-level overview of complex diseases.

**Figure 1:**
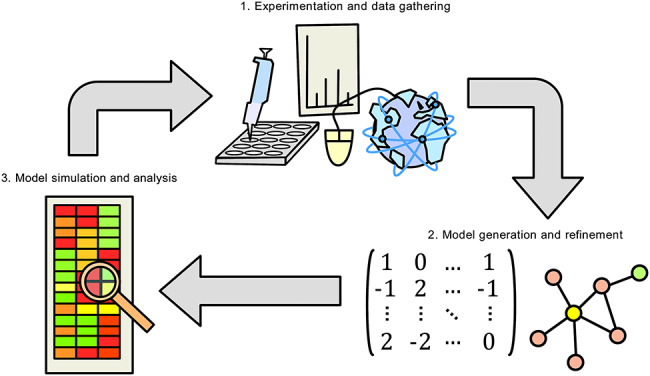
Iterative computational biology workflow. Data are gathered by experimentation, from the literature or from publicly accessible databases. Computational models describing biological knowledge are generated and refined. Models are used for *in silico* simulation, re-refinement of the model and hypothesis generation. Findings are validated experimentally, feeding into new data for the next iteration of the cycle.

Systems medicine has demonstrated concordance with previously published findings and can be used as a platform for *in silico* simulation, hypothesis generation and rational drug development (Figure 1). One of the goals of systems medicine is to identify biomarkers for stratification and per-stratum or personalised treatment. In this article, we assess the readiness of the liver disease field to allow for personalised treatment regimes. We then review systems-based approaches currently in use to overcome the challenges with heterogeneity in complex diseases, namely, genome-scale metabolic models (GEMs) and integrated networks (INs), and discuss their value to the scientific community. Finally, we review the collective successes afforded by diverse systems biology methods in revealing common themes implicated in the progression of liver diseases including NASH/NAFLD and HCC, namely differential expression of pyruvate kinase muscle-type (PKM) isoform transcripts, differential acetate utilisation and differential regulation of redox metabolism. We therefore propose systems biology to be the best approach towards personalised treatment of complex liver diseases and believe that identifying stratifying and treatment markers will aid greatly in this pursuit.

## Liver disease and metabolism

### The altered metabolism in liver diseases

Common metabolic disorders are often complex in nature, involving strong multigene components, and containing many different underlying mechanisms that result in the same gross phenotype. These metabolic disorders often play a critical role in the pathogenesis of disease—for example, obesity has adverse effects on health and is associated with certain types of cancer [10], in addition to being strongly linked to NAFLD.

Other known traditional risk factors involved in the development of NAFLD include age, high blood pressure, high cholesterol, diagnosis of type II diabetes (T2D) and metabolic syndrome. NAFLD has also been diagnosed in people without any of the risk factors. A recent study focused on NAFLD patients and characterised patients according to liver fat content (high hepatic steatosis (HS) was defined as >5.5% liver fat percentage; low HS was defined as <5.5% liver fat percentage) and was able to suggest alternative treatment strategies for NAFLD patients [11], namely dietary supplementation of serine, L-carnitine, N-acetyl L-cysteine (NAC) and nicotinamide riboside (NR). These strategies were verified to positively improve the disease state in mice and humans [12]. The abnormalities in serine metabolism have also been previously reported based on the integration of proteomics and transcriptomics data in NAFLD patients [13].

The role of diet in NAFLD progression has previously been investigated in attempts to improve liver fat metabolism in NAFLD patients. Recently, an isocaloric low-carbohydrate diet was found to be beneficial for NAFLD patients [14]. This disease results in excessive release of free fatty acids into the bloodstream, which is associated with metabolic syndrome. The intervention with an isocaloric carbohydrate-restricted diet was found to induce improved fatty acid oxidation, in addition to inducing decreased glycolytic and tricarboxylic acid (TCA) fluxes, which in turn would lead to reduced fatty acid biosynthesis (FAB). Previous studies have also suggested that the supplementation of the diet with natural substances may lead to a decrease in the level of the fat accumulated in the liver (e.g. L-carnitine can activate fatty acid uptake) and hence be used for the treatment of NAFLD [14].

Classically, patients with risk of liver disease may mitigate their personal risk through lifestyle changes such as exercise and weight loss over extended periods of time. Interestingly, a longitudinal study in weight gain and loss in insulin-resistant (IR) and insulin-sensitive (IS) individuals revealed health implications of weight gain that were immediately reversed or not immediately reversed after weight loss [15]. For example, genes associated with inflammatory response and dilated cardiomyopathy were activated following weight gain, but only inflammatory response genes decreased back to baseline with weight loss.

The high metabolic activity of the gut microbiome has to be considered when investigating food intake by the human body in health and disease states. For example, gut microbiota has been found to affect host amino acid metabolism, with substantial modifications in glutathione metabolism occurring in the liver and other metabolically active tissues [16]. Previous associations have also been identified between the composition of the gut microbiota and development of various complex diseases, including NAFLD and various cancers [17]. Furthermore, recent metagenomic studies have provided additional novel insights into metabolic diseases and are able to identify possible therapeutic targets [18]. These findings should be able to facilitate personalised interventions based on metagenomics analysis. The aforementioned longitudinal study also revealed disease case—specific changes in the microbiome—in particular, it was found that following weight gain, the level of the mucin-degrading bacterium *Akkermansia muciniphila* increased significantly in the stool samples of IS individuals, but not IR. This finding is consistent with previous animal-based studies suggesting that the bacterium confers protective properties against insulin resistance [19, 20].

### Modulation of liver metabolism using natural substances

With the motivation of addressing specific metabolic disorders, natural substances have been shown to restore normal hepatic lipid metabolism and reduce HS in NAFLD patients. As reviewed recently [12], a three-step strategy involving increased mitochondrial fatty acid uptake, increased mitochondrial fatty acid oxidation and increased availability of glutathione (GSH) was predicted to correct liver metabolism altered in NAFLD, in particular identifying the metabolic cofactors serine, carnitine, NAC and NR by using network modelling [11]. Such natural substances can form the basis of per-stratum or personalised therapies for patient groups identified by stratification. Several therapies using natural substances have been used in clinical trials for the treatment of NASH/NAFLD and HCC as presented in Table 1, and select examples are described below.

**Table 1:** Survey of current and completed clinical trials using natural substances to treat liver diseases

NCT identifier	Treatment and dosage (if specified)	Conditions	Phase	Status
NAFLD/NASH				
NCT03073343	Betaine 2 g or 4 g twice daily	NAFLD/T2D	N/A	Recruiting
NCT01016418	Bovine colostrum powder 600 mg three times	NAFLD/NASH	Phase 1/2	Completed
NCT02929901	Caffeine 200 mg daily; and/or chlorogenic acid 200 mg daily	NAFLD/T2D	Phase 2/3	Completed
NCT02458586	Canola oil 50 g daily	NAFLD/obesity/prediabetes/ dyslipoproteinemia	N/A	Unknown
NCT01707914	Chinese bayberry juice 250 mL twice daily	NAFLD	N/A	Completed
NCT03375580	Compound zhenzhu tiaozhi 4 tablets three times daily; or metformin 0.5 g three times daily; or simvastin 20 mg daily	NAFLD	N/A	Recruiting
NCT02908152	Curcumin 1500 mg daily	NAFLD/T2D	Phase 2/3	Unknown
NCT01934777	DHA 250 mg, vitamin E 39 UI, choline 201 mg daily	NAFLD/fibrosis/obesity/ MetS	Phase 3	Completed
NCT00820651	Diamel® 660 mg every 8 h	NASH/IR	Phase 3	Completed
NCT01936779	EPA/DHA 4 g daily	NAFLD	N/A	Completed
NCT03260543	Fermented ginseng powder 125 mg or 500 mg daily	NAFLD	N/A	Completed
NCT00681408	Fish oil 3 g daily	NAFLD/NASH	Phase 2/3	Completed
NCT00230113	Fish oil 4 g daily; or safflower oil 4 g daily	NAFLD	Phase 2	Completed
NCT01547910	Fish oil 400–1200 mg	NAFLD	Phase 2	Completed
NCT02395900	Flaxseed powder 30 g	NASH	Phase 2/3	Completed
NCT03625284	FucoVital® (microalgae fucoxanthin extract)	NAFLD	N/A	Not yet recruiting
NCT02535195	Ginger supplement (ginger 500 mg) 2 capsules twice daily	NAFLD	Phase 2/3	Completed
NCT01553500	Glucomannan 5 g daily	MetS/NAFLD/IR	Phase 2	Completed
NCT03801577	Hepaxa® (EPA/DHA) 4 capsules daily	NAFLD/NASH	N/A	Not yet recruiting
NCT03377140	Hesperidin 2 capsules	NASH	N/A	Unknown
NCT03377153	Hesperidin 2 capsules, flaxseed 30 g	NAFLD/NASH	N/A	Unknown
NCT03734510	Hesperidin supplement 2 capsules; and/or flaxseed 30 g	NAFLD/NASH	N/A	Recruiting
NCT00816465	*Hoodia gordonii* extract 1 tablet daily	NAFLD	Phase 1	Completed
NCT02992470	Hydrolysed oyster extract 250 mg three times daily	NAFLD	N/A	Unknown
NCT03914495	Inulin 10–40 g daily	NAFLD	N/A	Recruiting
NCT02642172	Inulin-type fructan (inulin/oligofructose 75/25) 16 g daily	NAFLD/MetS	N/A	Recruiting
NCT00586885	L-Alanine 6 g one to three times daily	NASH	N/A	Completed
NCT03439917	L-Carnitine tartrate 2 g, Slimfast® 325 ml twice daily	NAFLD/IR	N/A	Recruiting
NCT03463967	Lycopene-enriched tomato juice 100 g daily	NAFLD	N/A	Recruiting
NCT03135873	Mastiha 2.1 g daily	NAFLD	Phase 1	Recruiting
NCT02647294	Maxicor® n-3 PUFA 3.6 g daily	NAFLD	N/A	Active, not recruiting
NCT01940263	Medox® anthocyanin 320 mg daily	NAFLD/NASH	Early Phase 1	Completed
NCT03864783	Meriva® curcumin supplement 1000 mg twice daily	NAFLD/obesity/IR/glucose tolerance impaired	N/A	Not yet recruiting
NCT00063635	Metformin 500 mg daily; or vitamin E 400 IU twice daily	NAFLD	Phase 3	Completed
NCT03942822	Milled chia seeds 25 g daily	NAFLD	N/A	Completed
NCT01056133	n-3 PUFA 1.0 g (EPA/DHA 0.82/0.44 g) daily	NASH/NAFLD	Phase 2	Completed
NCT01285362	n-3 PUFA 4.0 g (EPA/DHA 465/375 mg per 1 g capsule) daily	NAFLD	N/A	Completed
NCT02117700	NAC 600 mg once or twice daily	NAFLD/obesity/CVD	Phase 2	Unknown
NCT03850886	Nature’s Life® niacinamide supplement 1000 mg daily	NAFLD	Phase 2	Recruiting
NCT02307344	*Nigella sativa* 1 g twice daily	NASH	N/A	Unknown
NCT03838822	NR 1 g, L-carnitine 3 g, serine 20 g, NAC 5 g	Healthy	Early Phase 1	Completed
NCT02369536	Nutraceutical mixture [fish oil (DHA 70%), phosphatidylcholine, silymarin, choline bitartrate, curcumin, D-α-tocopherol; choline 82.5 mg] 1600 mg daily	NAFLD	N/A	Completed
NCT02923804	Omega-3 supplement 3 g daily	NAFLD	N/A	Completed
NCT02201160	Omega-3 supplement 4 capsules daily	NAFLD	Phase 1/2	Unknown
NCT03132662	Optifast® (0.35 g linolenic acid) 1 serving, four times daily; or Oceano3® Krill Oil (EPA 150 mg, DHA 90 mg) 1000 mg three times daily	Obesity/NAFLD/NASH	N/A	Not yet recruiting
NCT01875978	Phytosterols 1.8 g daily	NAFLD	N/A	Completed
NCT01002547	Pioglitazone 30–45 mg daily, vitamin E 400 IU twice daily; or vitamin E 400 IU twice daily	NASH	Phase 4	Completed
NCT03627819	Plant sterols 3 g daily; or plant stanols 3 g daily	NAFLD	N/A	Recruiting
NCT00977730	Protandim 1675 mg daily	NASH	N/A	Completed
NCT00870077	ProWHEY® 94 CFM/SponserR® 20 g three times daily	NAFLD/obesity	N/A	Completed
NCT03047668	PUFA	T2D/NAFLD/obesity/ dyslipidemia/ hypertension/MetS	N/A	Unknown
NCT01992809	PUFA (ALA 64%, EPA 16%, DHA 21%) 945 mg three times daily	NAFLD	Phase 3	Completed
NCT00819338	PUFA 5 g daily	NAFLD	Phase 2	Completed
NCT02030977	Resveratrol 1 capsule daily	NAFLD	Phase 2/3	Completed
NCT01446276	Resveratrol 500 mg three times daily	NAFLD/Obesity	N/A	Completed
NCT01464801	Resveratrol 500 mg three times daily	NAFLD	N/A	Completed
NCT02216552	ResVida® resveratrol 75 mg twice daily	NAFLD/T2D/MetS	Phase 2/3	Completed
NCT02568787	Rice bran arabinoxylan compound 1 g twice daily	NAFLD	N/A	Completed
NCT02599038	Serine daily	NAFLD/NASH	Phase 1/2	Completed
NCT01650181	Siliphos® 140 mg, selenium 15 μg, methionine 3 μg, α-lipoic acid 200 mg twice daily	NAFLD/NASH	Phase 4	Completed
NCT03749070	Silymarin 700 mg, vitamin E 8 mg, phosphatidylcholine 50 mg daily	NAFLD	N/A	Recruiting
NCT03319199	Slim Water® (L-carnitine 2000 mg, magnesium 150 mg) 1 serving daily	NAFLD/NASH	N/A	Not yet recruiting
NCT01956825	Slim Water® (magnesium lactate 150 mg, L-carnitine 2000 mg)	NAFLD/NASH	Phase 4	Unknown
NCT03664596	Sublimated mare milk 1 sachet three times daily; with/without UDCA capsule 250 mg two or three times daily	NASH	N/A	Recruiting
NCT03738358	Trehalose 5 g daily	NAFLD	N/A	Completed
NCT01511523	Vitamin C/silymarin/carnitine 3 capsules twice daily	NAFLD/NASH	N/A	Unknown
NCT03084328	Vitamin D 2000 IU daily	NAFLD	N/A	Completed
NCT01623024	Vitamin D 20000 IU weekly	NAFLD	Phase 3	Unknown
NCT02132442	Vitamin D 50000 IU weekly	T2D/NAFLD/Vitamin D deficiency	Phase 3	Completed
NCT01571063	Vitamin D3 2100 IU daily	NASH	Phase 2	Completed
NCT02962297	Vitamin E 100 mg three times daily	NASH	N/A	Active, not recruiting
NCT01792115	Vitamin E 200 IU or 400 IU or 800 IU daily	NAFLD	Phase 2	Completed
NCT00063622	Vitamin E 30 mg daily; or pioglitazone 800 IU daily	NASH	Phase 3	Completed
NCT02690792	Vitamin E 400 IU twice daily	NAFLD/NASH	N/A	Completed
NCT00655018	Vitamin E 600 IU, vitamin C 500 mg daily	NAFLD/inflammation/ fibrosis/IR	Phase 2/3	Completed
NCT03669133	Vitamin E 800 IU daily	NAFLD/NASH/HIV	Phase 2	Recruiting
NCT03988725	Vitamin E 800 IU daily	NASH/HIV mono-infection	N/A	Completed
NCT00509418	Viusid 1 sachet three times daily	NASH	Phase 3	Completed
NCT02983669	*Zataria multiflora Boiss.* 350 mg twice daily	NAFLD	N/A	Completed
NCT02178839	β-Glucan oat supplement 8.5 g daily	NAFLD/NASH	N/A	Unknown
Liver cirrhosis				
NCT03285217	Abbott Nutrition® 1 serving, vitamin D 160 IU twice daily	Liver cirrhosis/sarcopenia/malnutrition	N/A	Active, not recruiting
NCT02132962	Amino acid infusion	Liver cirrhosis	N/A	Completed
NCT02023229	BCAA	Liver cirrhosis	Phase 4	Completed
NCT00931060	BCAA 0.45 g/kg daily	Liver cirrhosis/hepatic encephalopathy/hepatic insufficiency	N/A	Completed
NCT00955500	BCAA 30 g (leucine 13.5 g, isoleucine 9 g, valine 7.5 g) daily	Liver cirrhosis/hepatic encephalopathy	Phase 4	Completed
NCT03339232	Bulk Supplements® BCAA powder (L-leucine 50%, isoleucine 25%, valine 25%) 1788 mg seven times daily	Liver cirrhosis	N/A	Recruiting
NCT03605147	Calcium-HMB 1.5 g twice daily	Liver cirrhosis/sarcopenia	N/A	Recruiting
NCT03354299	Coconut milk 50 mL daily	Liver cirrhosis/malnutrition	N/A	Completed
NCT03908255	Do Vitamins® BCAA supplement	Liver cirrhosis/liver failure/HCC	Phase 2	Not yet recruiting
NCT02650245	EAS Myoplex® protein drink, lactulose 10 g	Liver cirrhosis	N/A	Completed
NCT02407769	Enterex® Hepatic bag (BCAA 8.63 g) 1 serving daily	Liver cirrhosis	N/A	Unknown
NCT00168961	Fresenius Kabi supplement	Liver cirrhosis	Phase 4	Completed
NCT03080129	Fresubin® Energy 200 mL daily	Liver cirrhosis/sarcopenia	N/A	Recruiting
NCT03503708	Herbal supplement (*Phyllanthus niruri*, *Boerhavia diffusa*, *Picrorhiza kurroa*) two capsules twice daily	Alcoholic liver cirrhosis	N/A	Not yet recruiting
NCT03234920	HMB 1.5 g twice daily	Liver cirrhosis/sarcopenia	N/A	Completed
NCT03892070	HMB 1.5 g twice daily	Liver cirrhosis/sarcopenia	N/A	Recruiting
NCT02249741	Ibandronic acid 150 mg monthly	Liver cirrhosis	Phase 4	Completed
NCT01113567	Lactose-free milk (lactose 3.5 g); or whole milk (lactose 24 g)	Liver cirrhosis/hepatic encephalopathy	N/A	Suspended
NCT01773538	Lactulose 25 mL, three times daily, rifaximin 550 mg twice daily, Bramino® BCAA 30 g daily	Liver cirrhosis/hepatic encephalopathy	N/A	Completed
NCT01060813	Leucine supplement 10 g daily	Liver cirrhosis	N/A	Completed
NCT03208868	Leucine-enriched essential amino acids	Liver cirrhosis	N/A	Recruiting
NCT01408966	Lindt Excellence® 85% Cocoa dark chocolate 0.55 g/kg daily; or Lindt Excellence® Natural Vanilla white chocolate 0.63 g/kg daily	Liver cirrhosis/portal hypertension	Phase 2	Completed
NCT01894867	Magnesium	Liver cirrhosis	Phase 4	Unknown
NCT02321202	Omega-3 parenteral nutrition (Structolipid® 20%, Omegaven® 10%) daily	Liver cirrhosis/liver cancer	Phase 4	Unknown
NCT01260012	Praziquantel® daily; with/without antioxidant supplement daily	Schistosomiasis/liver fibrosis/periportal fibrosis/oxidative stress	N/A	Unknown
NCT01634698	Retinyl palmitate 1500 IU or 2500 IU once	Chronic liver disease	N/A	Completed
NCT00212186	Selenate (selenium 200 μg) daily; or selenomethionine (selenium 200 μg) daily	Liver cirrhosis	N/A	Completed
NCT02321579	Vitamin B6 50 mg daily; and/or glutathione 500 mg daily	Liver cirrhosis/liver cancer	N/A	Unknown
NCT02009748	Vitamin D 2800 IU daily	Liver cirrhosis/Vitamin D deficiency	Phase 2	Completed
NCT01463735	Vitamin E 350 mg twice daily	Liver cirrhosis	Phase 2	Completed
NCT00502086	Viusid® three sachets daily	Liver cirrhosis/chronic hepatitis C	Phase 3	Completed
NCT00312078	Yogurt 170 g twice daily	Liver cirrhosis/minimal hepatic encephalopathy	N/A	Completed
NCT02475928	Zinc gluconate 100 mg	Liver cirrhosis/dysgeusia	N/A	Recruiting
NCT02072746	Zinc sulfate 220 mg daily	Alcoholic liver cirrhosis	N/A	Unknown
Liver cancer				
NCT00945568	Aminoleban® EN (amino acids 6.5 g) 50 g twice daily	HCC/Chronic liver disease	N/A	Completed
NCT02327819	BCAA supplement 12 g daily	Primary liver cancer	N/A	Unknown
NCT01018381	BioBran® Arabinoxylan Rice Bran 1 g daily	HCC/hepatitis B	N/A	Unknown
NCT01666756	Chinese herbal formulation PHY906, sorafenib tosylate	Adult primary HCC/advanced adult primary liver cancer/advanced adult HCC/BCLC stage B adult HCC/BCLC stage C adult HCC	Phase 1	Active, not recruiting
NCT03908255	Do Vitamins® BCAA	HCC/cirrhosis/liver failure	Phase 2	Not yet recruiting
NCT00168987	EPA	Colorectal neoplasms/ HCC/cholangiocarcinoma	Phase 4	Completed
NCT01434524	LIVACT® (amino acids 13.0 g)	Liver cancer	N/A	Completed
NCT01392131	Oncoxin® syrup 25 mL, Oncoxin® 1 capsule twice daily	HCC	Phase 1/2	Unknown
NCT02041871	Oral Impact® powder 74 g three times daily	Hepatectomy/elective hepatectomy/malignant tumours	N/A	Completed
NCT00040898	Sho-saiko-to	Liver cancer	Phase 2	Completed
NCT01964001	Vitamin B6 50 mg daily; and/or coenzyme Q10 300 mg daily	HCC	Phase 2/3	Completed
NCT02321579	Vitamin B6 50 mg daily; and/or glutathione 500 mg daily	Liver cirrhosis/Liver cancer	N/A	Unknown
NCT01542281	Whey protein, dietary supplements	Colorectal neoplasm/biliary tract neoplasm/liver neoplasm	N/A	Unknown

ALA, α-linoleic acid; BCLC, barcelona clinic liver cancer; CVD, cardiovascular disease; DHA, docosahexaenoic acid; EPA, eicosapentaenoic acid; HIV, human immunodeficiency virus; HMB, β-hydroxy-β-methylbutyrate; IR, insulin resistance; MetS, metabolic syndrome; T2D, type II diabetes; VDD, vitamin D deficiency.

Vitamin A metabolism in NAFLD and its putative role in the progression of liver disease have recently been reviewed [21]. Vitamin A is required for a number of important physiological processes, ranging from cell proliferation and differentiation to immune regulation, in addition to glucose and lipid metabolism. The liver plays a key role in the metabolism of vitamin A and harbours the largest body supply of vitamin A in hepatic stellate cells (HSCs), mostly as retinyl esters. Liver diseases, particularly those resulting in fibrosis and cirrhosis, have a profound impact on vitamin A storage and metabolism. An impaired liver triggers HSCs to activate and transdifferentiate to myofibroblasts, leading to a loss of hepatic vitamin A stores and thereby causing dysregulated lipid metabolism. Hence, vitamin A metabolites are key co-regulators of hepatic lipid metabolism and therapies have been targeted at re-establishing proper levels of vitamin A that may restore order to hepatic lipid metabolism in NAFLD [22].

Furthermore, vitamin E has been proposed as a treatment for NAFLD owing to its status as a potent antioxidant that has the ability to reduce oxidative stress in NAFLD, which is believed to play a crucial role in producing the lethal hepatocyte injury that is associated with NAFLD [23]. This is in part due to reactive oxygen species inducing the peroxidation of hepatic triglycerides (TGs) with the subsequent release of reactive aldehydes damaging mitochondrial components [24]. Oxidative stress has also been identified as a factor that disturbs endoplasmic reticulum (ER) folding capacity and increasing amounts of accumulating data have implicated the disruption of ER homeostasis in NASH development [25]. Hence, there is a need to focus on the therapeutic efficacy of vitamin E in NAFLD/NASH. However, clinical trials involving vitamin E administration have only shown modest improvement in liver biochemistries so far: results include modestly reduced alanine transaminase (ALT) levels in children with NAFLD [26], reduced ALT and aspartate transaminase (AST) levels in NASH [27], and reduced ALT, AST and γ-glutamyl transpeptidase (GGT) when combined with ursodeoxycholic acid (UDCA) [28].

Studies also indicate the potential benefit of omega-3 supplementation for NAFLD patients and show an association with metabolic disorders [29]. For instance, long-term daily administration of n-3 polyunsaturated fatty acid (PUFA)-enriched olive oil can decrease AST, ALT, GGT, TG and fasting glucose levels [30], in addition to markedly enhancing adiponectin levels compared with control [31]. When used to supplement an American Heart Association (AHA)-recommended diet, long-term daily PUFA supplements can decrease ALT, TG and serum tumour necrosis factor α (TNFα) levels, as well as liver fat content compared with an AHA-recommended diet alone [32]. In NAFLD associated with hyperlipidemia, daily intake of seal oil-derived PUFA can result in decreased ALT, TG and low-density lipoprotein (LDL) compared with control [33]. These observations indicate that the supplementation of the diet with omega-3 fatty acids can improve liver biochemical features in NAFLD patients and can be used in combination with recommended dietary changes.

Other studied supplements include carnitine: twice-daily supplementation with a recommended diet has been associated with biochemical amelioration such as in ALT, AST, GGT, high-density lipoprotein, LDL, total cholesterol and TG levels in NASH compared with diet alone [34]; serine: associated with decreased ALT, AST, TG and alkaline phosphatase in NAFLD [11]; NAC: twice-daily dosage has been associated with decreased ALT in NAFLD compared with the twice-daily dosage of vitamin C [35]; and branched-chain amino acids (BCAAs): long-term oral intake has been linked with preventing progression to liver failure in advanced cirrhosis patients compared with lactoalbumin and maltodextrins [36], and increased serum albumin as well as general health perception scores in decompensated cirrhosis compared with diet therapy alone [37].

Finally, within alternative therapies, one example of note is silymarin, an herbal remedy derived from milk thistle seed known for its antioxidant properties [38]. NAFLD patients receiving silymarin in combination with vitamin E displayed normalised ALT, AST and GGT levels over a course of 12 months [39]. Given that vitamin E therapy alone has resulted in only modest benefits, this study demonstrates that alternative therapies could potentiate the therapeutic benefits of mainstream medicine. Indeed, several clinical trials involving natural and alternative substances, such as fish oil, Chinese bayberry juice and oyster extract, are ongoing or completed (Table 1).

Taken together, it is clear that potential therapies are abundant, demonstrating the readiness of the field to prescribe single or multiple natural substances to liver disease patients in a personalised manner. However, since the therapy space for combinatorial treatments is impossible to be explored exhaustively in the clinic, systematic consideration of human metabolism as a model is now clearly required.

## Genome-scale modelling of liver metabolism

The shift in focus to human metabolism and its regulation when determining the molecular mechanisms of these complex diseases requires reconstruction of functional human metabolic models using a systems medicine approach. GEMs are very suitable for understanding mechanistic relationships between genotypes and phenotypes in addition to revealing the underlying mechanisms that may be responsible for a complex disease [40]. These models generally encompass different parts of metabolism and associated enzymes, thus enabling the study of such interactions in a holistic manner. This can prove extremely useful when targeting enzymes for disease treatment or identifying biomarkers for diagnosis through changes in metabolite concentrations [41].

Reconstruction of a GEM involves integrating the substrates and products, respective stoichiometric coefficients, directionalities, and compartmentalisation of every biochemical reaction catalysed by every enzyme in the cell or tissue type in question, followed by flux balance analysis (FBA) and definition of a biological objective function, such as maximising biomass production or minimising ATP consumption [42]. A number of computational tools for the development and application of GEMs, such as Metabolic Adjustment by Differential Expression (MADE) [43], Toolbox for Integrating Genome-scale metabolism, Expression, and Regulation [44] and Relative Metabolic Differences (RMetD2) [45], are publicly available.

The most comprehensive global reconstruction efforts of human metabolism are currently Recon3D and Human Metabolic Reaction database version 2.0 (HMR2)—these generic human GEMs containing more reactions, metabolites and genes than previously reconstructed [41, 46]. Hence, it is often employed to build cell/tissue-type GEMs using a task-driven model reconstruction (tINIT) algorithm [8], which combines cell-type-specific transcriptomics and proteomics with defined metabolic tasks that the generated model should be able to perform. An example of a cell/tissue-type GEM is *iHepatocytes2322,* a consensus functional GEM for hepatocytes, which was reconstructed manually by integrating the contents of previously published human hepatocyte GEMs [13]. It extends previous models of the liver by incorporating extensive information about lipid metabolism, which is necessary for studying the effects of excess lipids on the underlying molecular mechanism of NAFLD. The *iHepatocytes2322* GEM has been utilised to analyse transcriptomics data from NAFLD patients identifying new potential biomarkers and therapeutic markers [13]. More recently, this GEM has been used in conjunction with FBA to generate personalised, simulation-ready GEMs for NAFLD patients. This approach identified altered GSH and NAD+ metabolism as a prevailing feature in NAFLD and suggested a potential treatment strategy for NAFLD patients based on increased synthesis of GSH and increased oxidation of fat [11].

Aside from cell/tissue-type GEMs, cancer-specific GEMs, such as the HCC-specific GEM, have been reconstructed using HMR2 and the tINIT algorithm, thus providing insights about tumour progression and discovering anti-cancer drug targets through the use of personalised HCC models [8]. This study identified 46 antimetabolites (chemicals which disrupt metabolism by inhibiting the use of a metabolite) which were specific to individual patients and hence emphasises the need to stratify patients according to different metabolic profiles.

The mapping of high throughput datasets onto reconstructed GEMs allows for the analysis of metabolic conditions between two different conditions. An invaluable tool used in this mapping process is RMetD2, which has successfully integrated relative transcriptomics data into GEMs in several cases. RMetD2 differs from other tools as it sets gradient constraints, allowing expression changes to be evaluated over several steps rather than considering only the overall change in expression as in MADE. RMetD2 can also be applied without an objective function, allowing for modelling where no clear objective is defined. To illustrate one example, transcriptomics data obtained before and after a carbon-restricted dietary study were integrated into *iHepatocytes2322* to determine the metabolic differences that occurred in the liver over the course of the study [14]. The reaction associated with the triacylglycerol pool generation, and thus indicates the accumulation of liver fat, was among the reactions that were significantly downregulated. Furthermore, transcriptomics data have been integrated into the *HepG2* GEM when investigating the metabolic differences between wild-type and pyruvate kinase liver and red blood cell (PKLR) inhibited liver cancer cell line, using constraints and differentially expressed genes (DEGs) from a recent study [47]. RMetD2 suggested that the glycolytic reaction that converts glucose 6-phosphate to fructose 6-phosphate is classified as downregulated in the PKLR-inhibited *HepG2* cell line. This suggests a decreased glucose consumption at the beginning of the glycolytic pathway, and as expected, a decreased glucose consumption in PKLR-inhibited cells was observed in the experimental validation.

## INs for liver metabolism

**Figure 2:**
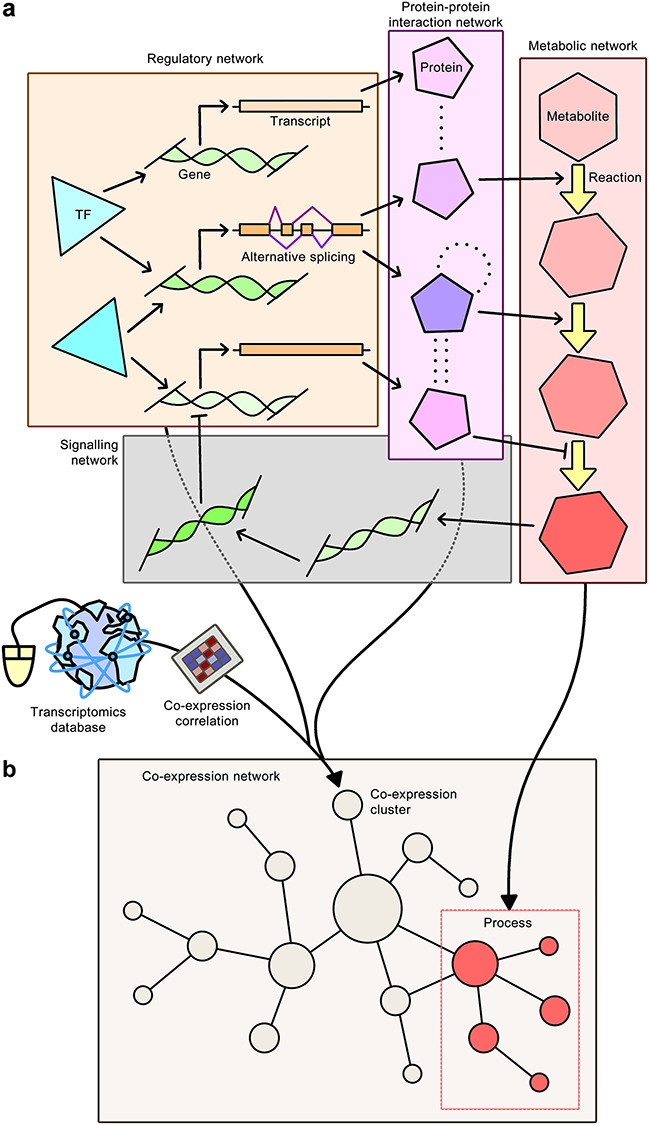
IN construction. The formation of INs and their overlap with CNs can reveal metabolic pathways that are regulated specifically in a tissue of interest. **A**, Formation of an IN through the integration of GEMs with other biological networks, including regulatory networks, PPINs and signalling networks. INs are necessary in order to cover the entire biological functions of cells and tissues in a holistic manner and should enable a better prediction of the cell phenotype. Arrows with barbed heads, activatory relationships; arrows with bars, inhibitory relationships; dotted lines, physical interactions; and arrows with filled heads, integration of data. **B**, Overlap of an IN with a CN can reveal tissue-specific functional and physical interactions, which can then be used to determine BPs that are uniquely regulated in a tissue of interest.

Further integration of biological knowledge into GEMs can be achieved through integration of GEMs with other biological networks, including transcriptional regulatory networks (TRNs), protein–protein interaction networks (PPINs) and signalling networks (Figure 2A). This integrative approach results in the formation of INs, which are necessary in order to cover the entire range of biological functions of cells and tissues in a holistic manner (GEMs cover only ~15–20% of all biological functions). Hence, these INs should enable a better prediction of the cell phenotype and may lead to a better understanding of how metabolic processes are altered when a certain enzyme is activated or inhibited. The Minimum Network Enrichment Analysis framework can also be applied to GEMs to generate all feasible alternative minimal networks, each of which corresponding to a distinct metabolic subsystem that can synthesise a target metabolite. This approach has previously been applied to investigate the deregulation of metabolic tasks in NAFLD and identified key regulators in different NAFLD phenotypes using transcriptomics data from liver samples [48].

The first attempt at generating an IN involved merging GEMs, TRNs and PPINs to generate cell-specific INs for hepatocytes, myocytes and adipocytes of lean and obese subjects [49]. This integration is performed by first combining sets of interactions in the TRN and PPIN, and then considering the enzyme-coding genes which overlap with GEMs. Network topologies provided by the cell-specific INs could then be employed to perform a co-regulation analysis for each metabolic pathway in the healthy and obese subjects. This approach was able to identify the dysregulation of fructose and mannose metabolism in obese subjects including plasma mannose levels increasing in response to obesity. Further associations were also found between plasma mannose levels and insulin resistance leading to the conclusion that mannose could be used to explain the variance in obesity-independent insulin resistance. Hence, this novel strategy of employing cell-specific INs had proven to be successful in identifying the dysregulation of biological functions in response to a disease, which in turn revealed the consequences on relevant metabolites in plasma and eventually led to the proposal of new candidate disease biomarkers. These findings prompted further studies [50] that also found elevated plasma mannose levels to be strong biomarkers for predicting future risk of several chronic diseases, including T2D, cardiovascular disease and albuminuria.

More recently, GEM, PPIN and TRN were merged to generate an IN for HepG2 cells, which could then be used to model the effect of inhibition of PKLR in these cells [47]. The findings suggested a global metabolic response to PKLR inhibition, including a decrease in glycolytic flux and FAB, both of which were experimentally validated, as later discussed in this review.

Overlap of INs with gene co-expression networks (CNs) can reveal tissue-specific functional and physical interactions, which can then be used to determine metabolic pathways that are regulated specifically in the tissue of interest (Figure 2B). An example of such an application includes the integration of TRNs, PPINs and CNs to identify liver-specific co-expression clusters, from which FASN–co-expressed genes (PKLR, PNPLA3, PCSK9) were identified as potential therapeutic targets for treating liver disease [51]. The database of tissue- and cancer-specific biological networks also employs a similar approach and has emerged as an invaluable tool towards gaining detailed insight into disease mechanisms, which in turn will lead to the development of efficient treatment strategies [52]. Human CNs were generated for 46 normal tissues and 17 cancers, and tissue-specific INs were generated for liver, muscle and adipose tissues through the integration of metabolic networks, TRNs and PPINs. Consequently, the overlap between functional and physical interactions provided by CNs and INs could be investigated, including functional relationships between genes and their relationships with biological functions. The comparative analysis of these networks may lead to the identification of tissue-specific targets that can be used to develop drugs that have minimum toxic effect on other tissues.

A top-down systems approach considering the interplay of interactions on many omics levels is preferred to gain a fuller insight into the global ramifications of perturbing a node in complex disease. A significant example was demonstrated in the HepG2 cell line, commonly used for the study of HCC. PKLR, a gene previously proposed by network analysis as a potential target for drug development [51], was inhibited *in silico* using an HepG2-specific IN reconstructed from an HepG2-specific TRN, GEM and PPIN [47] as well as RMetD2 for predictions in changes of fluxes. Simulations predicted the downregulation of pathways, including the TCA cycle, oxidative phosphorylation, FAB and fatty acid β-oxidation (FAO). In addition to these changes, the NADPH-generating folate cycle was predicted to be downregulated and the pentose phosphate pathway was predicted to be upregulated, in a metabolic flux shift away from the first half of glycolysis (glucose to fructose 6-phosphate (F6P) steps), to compensate for the depletion of NADPH. Interestingly, inhibition of PKLR was predicted to lead to increased flux in the second half of glycolysis [F6P to phosphoenolpyruvate (PEP) steps] despite PKLR itself being the enzyme responsible to convert PEP into pyruvate. Nonetheless, the simulated decreases in the first half of glycolysis and in FAB were validated by siRNA knockdown of PKLR in HepG2 cells. The knockdown experiments showed significant decreases in glucose uptake (down 40% compared with control) and adjusted total TG levels (down 15% compared with control). This study clearly demonstrates the benefits of network-based investigations in providing a deeper insight into the metabolic flux changes occurring in biological systems as well as directing hypothesis-driven research in the laboratory.

For various metabolic diseases, comprehensive collections of integrated clinical chemistry, anthropometric, plasma protein, metabolite and gut microbiome data have been generated in a number of longitudinal and cross-sectional studies [15, 53, 54]. Recent investigations that have integrated such omics data include an isocaloric low-carbon diet being found to be beneficial for NAFLD patients [14] and a novel glycine and serine deficiency phenotype being found in patients with NAFLD [11]. Hence, there is a need for resources and databases to investigate the associations between different types of omics data. In this context, the interactive database of multi-omics biological networks (MOBNs) [55] was created to provide a better framework to facilitate these types of investigations. It is highly expected that the integration of multiple omics data through the MOBN tool and other alternative tools may offer novel insights and provide a more extensive understanding of biological functions in the human body.

## Systems biology case studies for stratifying liver disease patients

Traditional efforts to treat disease through the development of drugs are generally directed by a small number of links associating the drug target with disease on the molecular or genetic level. Although useful for treating less complex medical complaints, these simple, single-layer associations are insufficient in explaining complex diseases, which require stratification into subclasses of disease. Biological networks have been invaluable in identifying underlying mechanisms driving subclasses of complex disease. Due to the global overview possible only by these systems-levels investigations, common fundamental pathways, genes and analytes have been identified for the stratification of patients or therapeutic targeting in multiple independent investigations. This, along with existing knowledge of the factors involved, demonstrate more confidently the accuracy of the results emerging from systems approaches for the generation of hypotheses to be tested at the bench or for rational drug development. Here, we summarise the corroborating findings of recent systems-level investigations involving identification of strata of disease, implicating network topology, acetate utilisation, isoforms and alternative splice products of PKM, and redox metabolism as important players in heterogeneous HCC.

### Stratification of HCC patients based on network topology

Networks integrating multi-omics data have also shown to be more effective than DEGs alone in stratifying individuals of complex disease into clusters with distinct biological or clinical profiles. It has been demonstrated that by generation of personalised functional gene-gene networks (fGGNs) for 369 individuals with HCC and 50 matched non-cancer individuals, fGGNs corresponding to HCC could be clustered to the exclusion of the non-cancer samples, a result not recapitulated when considering gene expression data alone due to the large heterogeneity among the HCC patients [56]. Integration of patient-specific transcriptomic data and an HCC-specific GEM was all that was required to elucidate the clustering, leading to the characterisation of the three proposed GEMs as described above (*iHCC1*, *iHCC2* and *iHCC3*).

Identifying stratifying genes or therapeutic targets based on network characteristics is an exciting emerging strand of systems biology that has already proven highly useful to researchers. By using network controllability theory, minimum driver set (MDS) nodes—those nodes required to achieve full control over a network [57]—and indispensable nodes—those nodes whose removal from the network increases the MDS [58]—can be identified. In a proof-of-concept study [59], personalised GEMs were constructed, and biomass production and ATP consumption were defined as objective functions for HCC GEMs and adjacent non-cancer GEMs, respectively, and functionality was determined based on whether the models could perform 57 and 56 previously documented metabolic tasks [8], respectively. Based on *in silico* gene silencing within these parallel models, eight genes were found to inhibit growth in all HCC GEMs, while at the same time inducing no change in non-cancer GEMs. Furthermore, three of these genes [protein kinase cAMP-activated catalytic subunit alpha (PRKACA), phosphatidylglycerophosphate synthase 1 (PGS1) and cardiolipin synthase 1 (CRLS1)] were identified as MDS nodes in HCC networks but not in non-cancer networks, indicating that inhibition of these genes would not be toxic to normal cells. Indeed, siRNA knockdown of these three genes in HepG2 and HepB3 cells led to promising reductions in cell growth by up to 35% in at least one cell line.

### Stratification of HCC patients based on acetate utilisation

**Figure 3:**
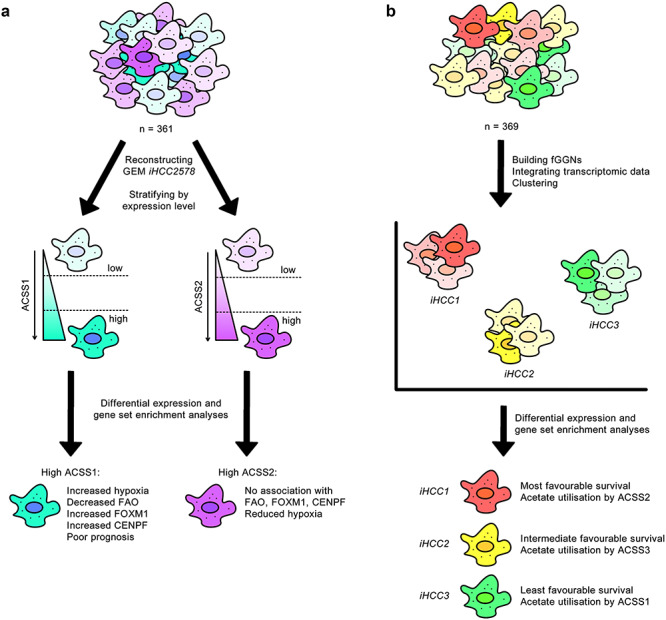
Independent studies highlight convergent conclusions in acetate utilisation in HCC heterogeneity. Separate investigations associated increased expression of ACSS1 with poor survival outcome. **A**, Stratification of tumours based on ACSS1 and ACSS2 expression led to the identification of poor prognosis markers in tumours expressing ACSS1 at a high level [6]. **B**, Clustering of tumours on the basis of fGGN and transcriptomic data resulted in the characterisation of three HCC subtypes, of which the subtype conferring the least favourable survival was found to preferentially express ACSS1 for acetate utilisation [56].

Several studies employing multi-omics network analysis approaches have been congruent in identifying major pathways contributing to liver disease. For instance, the genes encoding the enzymes catalysing the conversion of acetate to acetyl-CoA—namely mitochondrial enzymes ACSS1 and ACSS3, and cytosolic enzyme ACSS2—have been identified as stratifying genes in two independent network analysis studies (Figure 3).

In the first study [6], a reconstructed GEM for HCC, known as *iHCC2578*, predicted an unusually tightly regulated FAB pathway in a background of poorly or deregulated metabolic pathways, as normally expected in cancer. On the basis that the ACSS enzymes can generate acetyl-CoA to be used as a substrate for FAB, the authors stratified 361 HCC tumours by ACSS1 and ACSS2 expression level, separately, and found that high ACSS1 expression was linked to hypoxia, suppression of fatty acid oxidation, co-expression with the proliferation-specific transcription factor (TF) Forkhead box M1 (FOXM1) and centromere protein F (CENPF)—the implications of both of which in HCC are already established [60, 61]—and a poor prognosis for the patient. In contrast, no such associations were drawn between high ACSS2 expression and FOXM1 or CENPF, and in fact, a negative correlation could be drawn between high ACSS2 and hypoxic response (Figure 3A).

A more recent study [56] has also enabled tumour stratification by classifying personalised HCC GEMs into one of three HCC subtypes (*iHCC1*, *iHCC2* and *iHCC3*)—each of which have distinct gene expression, biological process (BP) and clinical survival characteristics. The reconstruction of cancer GEMs differs from non-cancer GEMs of the same cell/tissue type by having the formation of biomass as an additional metabolic task to ensure cell growth. The study stratified 369 HCC tumours into three clusters on the basis of an fGGN for HCC and patient transcriptomic data: *iHCC1*, indicating the most favourable survival; *iHCC2*, indicating intermediate survival; and *iHCC3*, indicating the least favourable survival [56]. In agreement with the prognostic characteristics of the ACSS1 and ACSS2 enzymes as described above, it was seen in HCC subtype-specific GEMs that *iHCC1* tumours favourably expressed ACSS2, *iHCC2* tumours ACSS3, whereas *iHCC3* tumours ACSS1 for acetate utilisation (Figure 3B). This concordance between independent studies highlights acetate utilisation as a key area of interest for the stratification and possibly treatment of patients suffering from HCC.

### Pyruvate kinase isoform expression profiles can inform cancer survival rates

PKM expression has been strongly associated with cancer survival, but the direction of the correlation is contradictory among different tissues of the human body [62]. To illustrate this point, high expression is an unfavourable prognostic marker for liver HCC, pancreatic adenocarcinoma, head and neck squamous cell carcinoma and lung adenocarcinoma; however, it is a favourable prognostic marker for other cancers such as kidney renal clear-cell carcinoma (KIRC), skin cutaneous melanoma, stomach adenocarcinoma and thyroid carcinoma. This heterogeneity can in part be explained by the fact that alternative splicing results in 14 isoforms of PKM, the major isoforms being PKM1 and PKM2, which differ by mutually exclusive exons 9 and 10 [63]. Therefore, contradictory treatment in the activation and inhibition of PKM has been suggested according to the type of cancer a patient has been diagnosed with.

To further investigate PKM transcripts at the functional level, the top and bottom quartiles of gene expression for each transcript in all cancers were compared in order to find DEGs, and this was followed by gene ontology (GO) enrichment analysis. The DEG/GO analysis identified two transcripts (ENST00000335181 and ENST00000561609), which includes the transcript for PKM2, associated with favourable survival in TCGA KIRC datasets. Two further transcripts (ENST00000389093 and ENST00000568883), associated with unfavourable survival, were also found. These opposite prognostic effects between the sets of transcripts were validated using an independent Japanese KIRC cohort of 100 patients [64]. This previous study confirmed that the former transcripts were associated with favourable survival, whereas high expression of the latter transcripts was associated with unfavourable survival. Thus, there was agreement between this independent KIRC cohort and the TCGA KIRC cohort.

**Figure 4:**
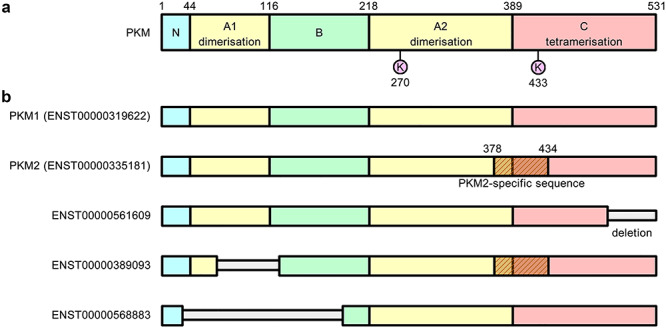
Alternative splice isoforms of PKM. Homology modelling and structure alignment can reveal functionally important sites and identify functionally significant deletions that occur in different PKM isoforms [62]. **A**, The template structure for PKM consists of four domains. The A-domain participates in the formation of dimers and the C-domain mediates the interactions between dimers that allow them to form tetramers. The active site (K270) and FBP binding site (K433) are shown. **B**, The alternatively spliced forms of PKM reveal large deletions corresponding to the ADP binding site in isoforms ENST00000389093 and ENST00000568883, which may impede dimerisation. In TGCA KIRC datasets, these transcripts are associated with unfavourable survival.

In an attempt to explain the differing survival rates between the sets of transcripts, the protein products of the prognostic transcripts were characterised and aligned with a template structure for PKM (Figure 4A). Amino acid sequence analysis revealed large deletions in the unfavourable survival transcripts, with ENST00000389093 and ENST00000568883 having deletions in the A1 and B domains (Figure 4B), which may impede dimerisation [65]. The amino acid alignment of translations of uncharacterised transcripts also revealed that ENST00000561609 had deletions in the C-terminal region, which may impede tetramerisation. Moreover, the region at residue range 389–433 more closely resembles PKM1 rather than PKM2 for isoforms ENST00000561609 and ENST00000568883. In this region, fructose 1,6-bisphosphate binds K433 (present in PKM2 but not PKM1), activating tetramer formation in PKM2 [66]. In contrast, PKM1 exists as a stable tetramer that has high constitutive activity [67].

Homology modelling and structure alignment with PKM1 and PKM2 revealed that ENST00000389093 lacked the catalytic site for ADP binding at residues 59–132 as a number of key contact residues within this range were missing. A newly ordered loop was found in place of the ADP binding site and it is unknown whether this loop can bind ADP in place of the active site. Meanwhile, ENST00000568883 had deletions in the A and B domains as well as the entire N-terminal domain, compared with PKM1 structure. Hence, the large deletions in the unfavourable survival transcripts corresponded to the ADP binding site, but it is still unknown whether these isoforms can bind ADP.

A further example of pyruvate kinase isoforms informing on cancer survival rates includes the aforementioned study on the fGGN-assisted stratification of HCC patients. Here, it was revealed that the gene expression in the poor-survival iHCC3 cluster was enriched for genes associated with cancer hallmarks compared with the good- and intermediate-survival iHCC1 and iHCC2 clusters [56]. In particular, PKM was identified as a potential stratifying gene for iHCC3: this cluster being associated with poor prognosis of HCC. Interestingly, it was seen that iHCC1 and iHCC2 cluster tumours use liver-specific PKLR for the utilisation of pyruvate rather than the muscle isoform PKM, signifying metabolic dysregulation on multiple pathways, indicative of more advanced or aggressive cancer.

### Dysregulated redox metabolism and hypoxia as hallmarks of HCC

It is known that imbalances in redox metabolism influence proliferation and tumourigenesis, thus making redox metabolism a potential target for cancer treatment. Hence, several recent efforts have targeted redox metabolism in cancer [68, 69]. In addition, a systematic examination of redox behaviour in HCC has been performed [70], which has allowed for a greater understanding of redox behaviour in HCC and its relationship with metabolism, signalling and patient clinical data.

**Figure 5:**
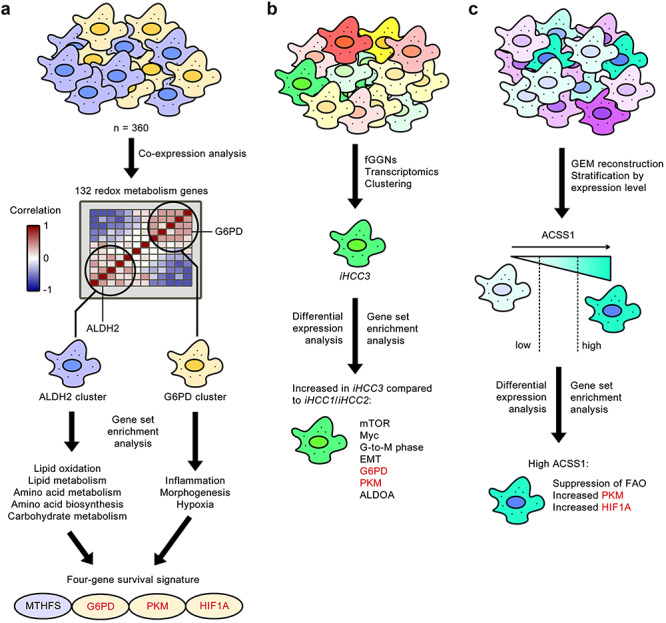
Independent studies reveal redox metabolism as a commonly dysregulated cellular function in heterogeneous HCC. Three separate investigations identified common redox metabolism genes (shown in red) as being associated with poor favourable survival of HCC. **A**, Stratification by antagonistic clusters of co-expressing redox metabolism genes reveals that the cluster associated with the least favourable survival is enriched for genes associated with inflammation, morphogenesis and hypoxia [70]. **B**, Differential expression between *iHCC3* and *iHCC1*/*iHCC2* tumours also identified elevated G6PD and PKM expression [56]. **C**, Differential expression of high ACSS1 HCC versus low ACSS1 HCC also revealed increased PKM and HIF1A [6].

This recent study stratified 360 HCC patients based on the expression of 132 redox metabolism genes identified two distinct clusters of redox genes. These two groupings, named the glucose 6-phosphate dehydrogenase (G6PD) cluster and the aldehyde dehydrogenase 2 (ALDH2) cluster based on the key genes existing in each, were found to be positively co-expressed with genes in the same cluster but negatively co-expressed with genes in the opposite cluster. ALDH2 cluster genes were enriched for GO BP terms such as lipid oxidation and metabolism, amino acid metabolism and biosynthesis and carbohydrate metabolism; however, G6PD cluster genes were associated with hallmarks of cancer-related functions [71] such as inflammation, morphogenesis and hypoxia. From the generation of cluster-specific GEMs for HCC, a four-gene signature consisting of PKM, folate metabolism gene MTHFS, G6PD and hypoxia-inducible factor 1 alpha (HIF1A) was proposed, indicating the activation of hypoxia response genes and the regulation of redox metabolism as targets of interest for the stratification and/or treatment of HCC patients (Figure 5A). HIF1A affects glycolytic genes, such as PKM, enabling them to cope with reductions in oxygen availability and consumption [72]. Enhanced expression of such glycolytic enzymes results in high rates of glycolysis. In cancer cell subpopulations, however, enhanced glycolytic flux and reduced oxidative phosphorylation can be achieved even in aerobic conditions [71], resulting in a change in metabolism known as aerobic glycolysis, or the Warburg effect [73], further contributing to cellular redox imbalance.

The fGGN-based HCC stratification study [56] showed that the differentially upregulated genes in *iHCC3* also included the mammalian target of rapamycin (mTOR), the oncogene Myc, genes involved in the G-to-M–phase progression of the cell cycle and genes involved in the epithelium-to-mesenchymal transition. In particular, redox metabolism genes were once again identified as potential stratifying genes for *iHCC3*: these included the aforementioned G6PD and PKM, as well as ALDOA (aldolase, fructose bisphosphate A), thus strengthening the association between redox metabolism and poor prognosis of HCC (Figure 5B).

In the differential expression analysis between high- and low-expressing ACSS1 HCC tumours [6], it was seen that high ACSS1 was associated with the suppression of FAO and increased PKM, a combination that has been previously linked to hypoxia and de-differentiation in HCC [74]. This, taken with the fact that HIF1A was found to be significantly positively co-expressed with ACSS1, suggests malignant growth under hypoxic response and a strong Warburg effect in cells highly expressing ACSS1 (Figure 5C).

Using diverse systems methods, three independent studies have separately converged to a conclusion implicating dysregulated redox metabolism and hypoxia as active hallmarks of cancer in subsets of HCC. Given that we have already highlighted similarities in the acetate utilisation of high ACSS1 tumours and *iHCC3* tumours, it is not inconceivable that these three independent subsets of HCC may not be mutually exclusive.

## Conclusion

The heterogeneity in complex diseases strongly indicates that personalised therapies are required for treatment through the stratification of the heterogeneous disease population. We highlight the recent progress made in context-dependent analysis of high-throughput data through reconstructed GEMs and give examples of how this approach has greatly contributed towards addressing the heterogeneity in liver diseases. The focus of our chosen examples revolves around the application of cancer-specific GEMs and biological networks in identifying key genes for stratifying and treating HCC. Namely, we identify acetate utilisation, PKM isoform expression and dysregulated redox metabolism as sources of HCC heterogeneity, identified across several independent systems-level studies. Hence, future studies should employ similar biological network analyses to identify additional sources of disease heterogeneity for the development of efficient stratification and treatment strategies for complex disease. In this effort, we illustrate the power of GEMs for modelling energy metabolism, INs for the integration of multi-omics data and utilising patient data (e.g., patient-specific transcriptomes) for the personalised treatment of HCC. Finally, novel methods for simulating the whole body functions should be developed analogous to a recent study that applied multi-scale, whole-systems models of liver metabolic adaptation to sugar and fat in NAFLD [75].

With 9.4 million patients with neurological disorders in 2015 [76] over 200 million patients with chronic kidney disease [76], and cardiovascular disease—the leading cause of deaths globally—causing over 17 million deaths each year worldwide [77], it is clearly of utmost significance to researchers to study more intensively the underlying causes of complex non-communicable diseases. Similar tools and methods have been successfully applied for the development of efficient treatment strategies for liver and other diseases, and the current growing library of natural therapeutic substances shows that the liver disease field is ready for personalised medicine.

**Key Points**

- Integrated multi-omics networks have been used to identify potential biomarkers and treatment strategies for the patients of complex liver diseases.
- Independent systems-level studies have yielded results that are consistent with one another as well as with previous knowledge.
- Systems biology could aid with hypothesis generation for the study of other complex diseases.

## Notes

^‡^Simon Lam and Stephen Doran are PhD students at King’s College London supervised by Professor Adil Mardinoglu. They are interested in the multi-omics modelling of neurodegenerative and cardiovascular diseases, respectively.

## Notes

**Simon Lam** Centre for Host-Microbiome Interactions, Faculty of Dentistry, Oral & Craniofacial Sciences, King’s College London, London, SE1 9RT, United Kingdom.

## Notes

**Stephen Doran** Centre for Host-Microbiome Interactions, Faculty of Dentistry, Oral & Craniofacial Sciences, King’s College London, London, SE1 9RT, United Kingdom.

## Notes

**Hatice Hilal Yuksel** Science for Life Laboratory, KTH - Royal Institute of Technology, Stockholm, SE-17121, Sweden.

## Notes

**Ozlem Altay** Science for Life Laboratory, KTH - Royal Institute of Technology, Stockholm, SE-17121, Sweden.

## Notes

**Hasan Turkez** Department of Medical Biology, Faculty of Medicine, Atatürk University, Erzurum, 25240, Turkey.

## Notes

**Jens Nielsen** Department of Biology and Biological Engineering, Chalmers University of Technology, SE-41296, Gothenburg, Sweden.

## Notes

**Jan Boren** Department of Molecular and Clinical Medicine, University of Gothenburg, The Wallenberg Laboratory, Sahlgrenska University Hospital, Gothenburg, Sweden.

## Notes

**Mathias Uhlen** Science for Life Laboratory, KTH - Royal Institute of Technology, Stockholm, SE-17121, Sweden.

## Notes

**Adil Mardinoglu** Centre for Host-Microbiome Interactions, Faculty of Dentistry, Oral & Craniofacial Sciences, King’s College London, London, SE1 9RT, United Kingdom; Science for Life Laboratory, KTH - Royal Institute of Technology, Stockholm, SE-17121, Sweden.
